# Humanization of care in forensic mental health wards: a qualitative study

**DOI:** 10.1080/17482631.2024.2448153

**Published:** 2024-12-30

**Authors:** Sylvie Anna Sollied, Monica Evelyn Kvande

**Affiliations:** a Department of Health and Care Sciences, Faculty of Health Sciences, UiT, The Arctic University of Norway, Tromsø, Norway; b Department for Postgraduate Studies, Lovisenberg Diaconal University College, Oslo, Norway; c Department of Anaesthesiology and Surgery, University Hospital of North Norway, Tromsø, Norway

**Keywords:** Forensic psychiatric care, humanizing, qualitative research, encounters, nursing

## Abstract

**Purpose:**

To explore nurses’ experiences related to humanizing care in high-security forensic units.

**Methods:**

This study has a qualitative design. Data consist of texts from study involved nine interviews with nurses working in two forensic mental health wards in Norway. The text was interpreted and analysed using Lindseth and Nordberg’s phenomenological—hermeneutical method.

**Results:**

Through an interpretation process, three themes emerged: “Openness and responsiveness to patients’ stories”; “Attention to patients’ individual needs”; and “Humanistic care in strict environments”.

**Conclusion:**

Nurses’ presence and caring attitudes in everyday forensic psychiatric care activities provide insights into individual patients’ lived experiences, vulnerability, fragility and tolerance limit. The ability to see and listen to patients at the human level and meet and respect patients in human fellowships is essential for care humanization. Additionally, the nurses in our study highlighted the need for nurses to feel safe and closely collaborate within professional healthcare teams.

## Introduction

Patient care in high-security forensic wards presents clinical, juridical and ethical challenges for healthcare professionals because of the fragility and vulnerability caused by severe mental disorders in patients. The patients’ lived experiences and the risks for recurrence necessitate forced hospitalization with risk assessments and high levels of security restrictions (Aga et al., 2019; Fosse et al., 2021). However, these patients are individual human beings in need of humanistic care despite their difficult life circumstances (Hörberg, 2018) and aggressive behaviour and pathology (Sanz-Osorio et al., 2023).

Forensic patients often have childhood experiences involving extensive maltreatment, parental criminality and substance abuse, parental violence against loved ones, and being raised in different foster placements (Fosse et al., 2021). Owing to this, Fosse et al. (2021) suggest that poor parenting with a lack of responsible and caring adults may explain patients’ increased stress and sensitivity, and the need for predictability and supervision requires an openness to cocreate individualized treatment and caring programmes in which patients are given the opportunity to explain and become involved in decision—making concerning their situations according to their own terms. Similarly, Kirkengen (2017) explain that persons who were exposed to neglect and abuse in childhood carry deeply embodied sensitivity; they have a strong sense of responsibility and need for control and are often very skilled at observing others and developing emergency strategies. Other studies have reported that considerable competence in staff is needed to accommodate patients’ emotional expressions and create trust-building therapeutic relationships (Hörberg, 2018; Norcross & Lambert, 2018; Oben, 2020).

Senneseth et al. (2021) reported that nurses are valuable sources of clinical information that may illuminate patients’ individual life experiences because of their competence in caring for patients in complex situations, which may elucidate challenges and opportunities in patients’ individual recovery processes. Nurses who act in a person-centred manner with an attitude that reflects their ethical values and working conditions that enable them to improve patient care may enhance the humanization of care (Sanz-Osorio et al., 2023). This valuable relational knowledge enables nurses to create safe surroundings, provides space for patients to find new ways of interacting with others and helps patients widen their tolerance limit in everyday life situations that enable genuine relationships and invite spontaneous conversations (Sollied et al., 2023).

Hammarström et al. (2022) reported that nurses became more involved and receptive to patients’ needs when they aimed to work in a patient-centred manner and were more compassionate and vulnerable with respect to their own feelings and safety needs.

Rytterström et al. (2021) argued that nurses and competence in healthcare staff have an impact on patients’ diagnosis and healing process through their presence on a 24-hour basis, and this aspect must be given more attention. In building therapeutic relationships with patients, having greater involvement and sharing experiences may empower patients and provide room for normality and independence without losing sight of the safety rules (Cartwright et al., 2022; Marklund et al., 2020).

To our knowledge, few previous studies have examined how nurses facilitate the humanization of care within forensic mental health in depth. Consequently, the aim of this study was to explore nurses’ experiences related to the humanization of care in high-security forensic wards.

## Materials and methods

### Design

A Ricoeur-inspired phenomenological—hermeneutic design that was adopted and further adjusted by Lindseth and Norberg (2022) was considered suitable for exploring nurses’ experiences related to the humanization of care in high-security forensic wards through in-depth interviews collecting data on experiences and essential meanings. The study was based on qualitative in-depth interviews with nine nurses working in two forensic care wards in Norway. The text was interpreted and analysed via the phenomenological—hermeneutical method inspired by Lindseth and Nordberg’s phenomenological—hermeneutical method.

To ensure a comprehensive report, the study was reported in accordance with the Consolidated Criteria for Reporting Qualitative Research (COREQ) (Tong et al., 2007).

### Procedure and setting

Approval for conducting the interviews was obtained from the head of the clinic. The participants were first informed and recruited via email and, after agreeing to participate, contacted by the first author via phone calls and SMS.

The wards are classified as having a high security level, anchored and regulated according to the Mental Health Act and the Penal Code as a regional security ward with national admission and staffed by a minimum of one healthcare professional per patient. The entrance halls to the wards have closed doors and metal detectors, and all staff carry keys and alarms. Patients’ have single rooms with cameras, and the wards have special alarm systems and equipment that characterize units with the highest level of security. The wards are staffed by psychiatrists and psychologists as heads of the department as well as head nurses as leaders of multidisciplinary teams with different healthcare professionals working 24-hour shifts.

### Participants and data collection

Participants were chosen through purposeful sampling based on their lived experiences with the study phenomenon; humanization of care in high-security forensic wards (Polit & Beck, 2020). Consideration was given the variations in sampling in terms of demographic characteristics (Table I). The inclusion criteria were nurses with a minimum of two years of experience working in forensic mental wards and providing care for forensic patients. Nine nurses from two different high-security forensic health care wards in a university hospital agreed to participate in the study.

**Table I.** Informant demographics.

Nurse	Gender	Age (year)	Education	Forensic ward experiences (years)
1	Male	39 y	Master in Health Sciences	4 y
2	Male	36 y	Bachelor of Nursing	6 y
3	Female	46 y	Master in Health Sciences	9 y
4	Male	31 y	Bachelor of Nursing	4 y
5	Female	36 y	Bachelor of Nursing	5 y
6	Male	44 y	Master in Health Sciences	11 y
7	Male	34 y	Bachelor of Nursing	5 y
8	Male	55 y	Bachelor of Nursing	7 y
9	Female	58 y	Bachelor of Nursing	8 y

In-depth interviews were conducted, recorded, and transcribed verbatim by the first author (SAS). The interviews were conducted in meeting rooms located outside the ward to ensure the participants’ anonymity and lasted from 60–120 minutes each.

To facilitate the nurses’ recollection of emotions and thoughts, a narrative interview form was used, and the opening question was as follows: “Could you tell me what you do in everyday activities to humanize care on the ward?” (Lindseth & Norberg, 2022). Follow-up questions, including “Can you please explain … ?” and “Can you please describe…?”, were asked for purposes of clarification and to avoid misunderstandings; these questions enabled the participants to put into words what, in their opinion, was the plot of their stories concerning their lived experiences and to participate in a process of telling and retelling their experiences using concrete, intelligible, meaningful, and loaded words (Lindseth & Norberg, 2022).

### Data analysis

The transcribed interviews were interpreted using the phenomenological—hermeneutic method of analysis developed by Lindseth and Norberg (2004, 2022), which consists of three methodological steps: a naïve reading, a structural analysis and a comprehensive understanding.

The first step involved a naïve reading of each text, which enabled the first author (SAS) and the last author (MEK) to obtain a naïve understanding of the meaning of lived experience of being nurses’ caring for patients in high-security forensic wards. In the subsequent steps, the text was separated into meaning units, which were later condensed and reflected upon to form themes. Structural analysis was a more precise component of the analysis, and multiple structural analyses could be conducted to reveal various meanings. The authors (SAS and MEK) reviewed the original text several times to ensure that every subtheme in the text could be identified and, conversely, that all relevant text was reflected in the themes. A comprehensive understanding was formed as part of the final step, which involved arriving at an understanding of the text as a whole and elucidating the expressed meaning of the participants’ words. This step was based on an interpretation of the naïve understanding and the findings of the structural analysis and focused on the research question, the context of the study and the relevant literature (Lindseth & Norberg, 2022).

### Ethical considerations

The study was conducted in accordance with the principles outlined in the World Medical Association Declaration of Helsinki (World Medical Association, 2013). The Norwegian Centre for Research Data (Sikt; reference number 27,841) and the research ethics committee at the hospital approved the study. All participants received written and oral information regarding the study aims in advance and were informed that participation in the study was voluntary. Written informed consent to participate was obtained from all participants before the interviews were conducted.

## Results

### Naïve understanding

Nurses are in charge of ensuring safety and supervising individual patients’ care and therapy within everyday life activities in wards. This position familiarizes nurses with how signs of change emerge in the social atmosphere and how individual patients experience hospitalization and a life with restrictions over the years.

Nurses’ awareness of how patients think and act by getting to know them as individuals may help patients express their needs, which is important for recovery and safety. If patients are telling nurses how they feel, the nurses could more easily understand and facilitate the avoidance of triggers and focus on meeting the patients’ recovery needs and providing humanized care.

### Structural analyses

The structural analysis resulted in three themes. The first theme was “openness and responsiveness to patients’ stories.” The second theme was “attention to patients’ individual needs.” The third theme was “caring in restrictive environments.”

These themes are illuminated in the following sections by using quotations from the nurses included in this study; the nurses are identified via numbers (e.g., Nurses 1–9, Wards 1–2).

### Theme 1: openness and responsiveness to patients’ stories

The theme of openness and responsiveness to patients’ stories reflects how nurses care for and engage patients who are seriously mentally ill and considered dangerous and what nurses do to practice caring in activities related to everyday life in wards. The nurses reported that working systematically to record observations of patients’ illness symptoms was important to share and reflect on within their multiprofessional team. In the nurses’ experience, a common understanding within the team and a calming atmosphere made them more aware of the importance of providing patient—centred care, and it helped them obtain a deeper understanding of individual patients’ trauma-related stressors. On the other hand, the nurses tried to lower the pressure from being consequently exposed to restrictions and limitations by signalling a common understanding of the patients’ rights by showing patients respect and understanding:

We are trained to appear calm and emotionally present as good role models in all kinds of social interactions in the ward. Similarly, we focus intensively on individual patients’ processes and try to understand traumatic experiences through attention to details that are important to the individual patient’s situation. We are concerned with trying to help internalize a perception in patients so that they can believe that other people usually have good intentions. We believe that a focus on securing and strengthening their human capacity helps them build resilience against stress and disease outbreaks in unexpected situations and can expand their individual tolerance limit. We invest in the relationship so that they will trust us so that we can understand how they feel and react in various contexts and so that we can gradually help them gain self-confidence and dare to trust in their own judgment. Nurse 1, ward 1.

The nurses reported that persons living with severe mental illnesses are aware of their own and others’ stigmatization and carry deep feelings of shame and self-blame. Being an inpatient for years also means that patients become deprived of internalizing societal skills and performance norms. The nurses focused on the patient’s life experiences and the problems that came from sparse contact with friends and families and a life spent in loneliness and isolation, which might explain patients’ self—accusing and self—focused ways of being. The nurses noted the importance of facilitating patients’ recovery by creating situations that may mirror normal life outside the hospital in a good way:

We try to balance the individual patient’s psychological and societal needs within the prescribed adaptation to the security rules, which in many ways deprive them from friendships, independence and the capability of making decisions in own life; otherwise, we maintain the situation that made them fragile in the first place. If they are upset, we give them space, such as following them to the room for a private talk or just sitting down and staying with them without talking and asking questions. If it is a good day, patients can choose what to do, such as going to a training studio, to a movie or to have a coffee in town. They are so self-centered and self-critical, so we try to counterbalance their fear of being misunderstood, humiliated, and disregarded by carefully pushing their tolerance limits and helping them experience how it feels to be accepted and appreciated by others. Nurse 2, ward 1.

The nurses in the study highlighted that forensic patients are individual persons and not a homogenic group and that it is important to become familiar with the individual patients’ lives to understand how each patient carries their own history and experiences. Furthermore, how patients interpret their surroundings and other people’s intentions and how this affects their ability to improvise or cope with common social expectations are important questions. The nurses must be able to contain patients’ frustrations and seek support from the professional team to find meaning and become aware of how their actions may influence patients’ behaviours in certain situations:

We consider that many of our patients have developed antisocial ways of being that we believe are strategies they needed to protect themselves in a challenging upbringing, and I am thinking of behaviors perceived by others in society as provoking, peculiar or dangerous. This means that the atmosphere at the ward is tense even if it is calm and quiet, so I am always alert and prepared if small changes occur; it can be something on the TV, a person saying something that triggers flashbacks or conspicuousness. If emotional turmoil occurs, we immediately clear up in misunderstandings; we stay with the patients and try to understand what is going on, and we must carefully try to reorient their interpretation to make them feel in control again. It is a main rule that those of us who work here show ourselves as people who can help patients cope with whatever comes up and that we do not fail but endure being tested and teased when difficulties occur to help patients sort them out, pull themselves together and gradually let go of trauma-related behavior. Nurse 3, ward 2.

### Theme 2: attention to patients’ individual needs

The theme of attention to patients’ individual needs describes how the nurses highlighted person-centred care by focusing on individual patients’ lived experiences and stories to understand their reactions and to help them avoid stress and confrontations. By spending time with and involving patients in decisions, the nurses could become aware of the patients’ lived experiences and understand how the patients reflected upon things and individual needs in their daily lives. The nurses’ intentions were to choose approaches that might provide patients with a sense of being listened to, respected, believed and trusted:

As far as I know, all the patients here have been sexually abused, but none of them will admit it or talk about it. It seems to be such an overwhelming shame for a grown man to open for and realize what happened to him in his past and to accept that this is a part of his story. In psychotic periods, patients may talk about violent sex in very upset and aggressive ways, and they may also talk to voices and respond with the body so that we understand that this is a trauma that is deeply embodied by and scary for them. In such situations, we do not intervene—we only try to show that we care and want to understand. We never push them to talk about it but accept that this is as far as they can go. Nurse 3, ward 1.

The nurses were concerned with investigating how patients carry their sorrows and losses with the intention of supporting patients in finding new ways to reflect on their situations and better ways of coping with their pasts. The nurses reported that focusing on individual needs is more than simply being kind towards patients and attentive to their problems. To be a person whom a patient finds trustworthy, the nurses must also aimed to create room for equality at the human level. At the same time, the nurses believed that a professional attitude is very important to ensure that patients can withstand and contain situations and keep the secrets that the nurses have been told:

I used to say that I care about him and that I expect a lot from him because he is a smart and clever guy who has managed and overcome very difficult life experiences and that he is a nice fellow that I like to spend time with. My experience is that most of our patients are extraordinarily obsessed with and often surprisingly precise and competent in their observations and predictions of other people’s intentions. I act professionally when facing the patients’ situations and reflect with them on how the health problems and happenings have affected their life and choices but also behave in a friendly and informal way to show the patients who I am and how I think as a person. In my experience, this is a balancing act that helps patients trust in me. Nurse 6, ward 1.

The nurses reported that receiving patients’ openhearted trust is very meaningful and satisfactory, but establishing a relationship of trust with patients often takes a long time. The patient´s daily form may vary from day to day, and taking care of patients’ needs for help is challenging in many ways:

I am trying to show the patient who I am a professional, but I am still concerned about talking and behaving as a friend joining another friend when we are outside the ward. I let the patient sort the tickets and books at the café, and if I must interrupt, I do it discreetly and use friendly humor as I do if I’m out with a friend. On the other hand, I am ensuring the patient that I am at their side all the time and praise them when we get back to the ward if they handle a situation effortlessly to illuminate progression if I know that they struggle with trusting own decisions because of fragile self-esteem. To put it short, I try to be a kind fellow who normalizes and facilitates a good experience without trivializing what they struggle with. Nurse 4, ward 1.

One nurse explained how he tried to balance restrictions and focus on illness recovery with care through establishing an equal relationship with patients within a professional setting:

I allow myself to show genuine empathy as well as enthusiasm on their behalf, and I believe it promotes equality and a mutual feeling of respect and care. I perform as a professional, and I express explicitly that I welcome and value considerations from the patients as part of their recovery process. I also act friendly if I have misunderstood a situation and go to the patient’s room afterwards to say I am sorry, and if a patient really scares me, I go and tell them how I feel about it when the turmoil is over. I use the opportunity to clear up as friends do and, in my experience, show that nurses are also vulnerable, as human beings invest in the relationship and illustrate normality. I believe it makes it easier to obtain shared opinions when we relate to situations in contexts that involve both of us. Nurse 4, ward 1.

The nurses claimed that working shifts helped them get to know the patients well—including their strengths and shortcomings—and that investing in this friendly relationship in daily life situations made it easier for the patients to open up to the nurses and enabled the nurses to help the patients expand their tolerance limits at their own pace over time:

To me, the strong belief that kindness and caring acts can help persons through severe illness and pain and bring out space for vitality and joy to persevere and withstand life challenges is a prerequisite for being a nurse here. Using your professional competence in everyday life situations to motivate fragile persons to open up and be honest about what they fear and then experience how they respond to respect and predictability—it makes me humble and so happy on their behalf. To see how humanity and care can make a person believe that a better future is possible, make me grateful and receptive to how life is from their point of view. Nurse 5, ward 2.

### Theme 3: humanistic care in strict environments

The nurses explained that as soon as the acute phase had passed and the patients were amenable for conversation and contact, they spent time with the patients with the intention of showing that they deserve to be respected and cared for like fellow humans.

Forensic care wards seem very restrictive, but the structured environment also gives a feeling of safety and predictability to us and to the patients. As nurses work shifts, we are positioned to represent normality and safety on a daily and consistent basis, and we practice individual follow-up as much as possible. This may be in the form of outdoor walks, time performing inspiring or relaxing activities, undisturbed conversations, and different social activities. To acquire knowledge about patients’ social codes and skills, we are concerned with showing that we have faith in patients and are interested in their competence and good qualities. We take them seriously, and we try to be role models who foster kindness and care for others. Nurse 6, ward 1.

The nurses reported that, within the safe and caring atmosphere at the ward, many patients significantly improved when they settled into the ward:

I am convinced that it is important to practice normality and to consistently support patients’ skills in everyday activities to help them cope with challenges and to show what it feels like to help each other and act in a friendly way. Although we have restrictive security and control systems, living rooms are furnished just like normal homes are, we follow normal life rhythms and routines and look after private and individual patient interests. Most patients appreciate that we always maintain predictability in routines, and we believe that it is calming and gives space for rest for us and for them. Nurse 8, ward 2.

The stability and predictability of everyday routines seemed to establish an atmosphere that provided foresight to everyone involved and an outer predictability that made patients’ diseases less prominent. The nurses explained this by illustrating how patients spontaneously express empathy and sympathy for others:

Our [nurses] main task is to secure safety at the ward, and to do this, we must also supervise the patients’ treatment programs by ensuring a correct rhythm in medication treatment, identifying severe side effects and creating space for individual care needs as much as possible. We also need continuous updates from psychologists and psychiatrists on processes that take place in individual psychoeducation so that we can be prepared for aftereffects and can understand and respond if a patient is upset or wants to continue talking about this with us afterward.

When all staff members have a common understanding and an attentive awareness of a patient’s situation, it seems to promote rest and coherence to the patients and enhance their recovery. We believe that patients gradually implement ordinary social norms and habits, which makes them more positive in judging other people’s intentions and more tolerant of their own situation. Even if patients seldom want to talk to and socialize with other patients on their own, they may show tolerance to fellow patients’ behavior if they have bad days and signal appreciation. The feeling of being accepted and cared for, eases the integration of humanity in relation to others, and it is touching to see. Nurse 9, ward 1.

The nurses reported that if they managed to give individual patients focused attention and spend time with them in different situations, the nurses themselves became more familiar with patients’ different daily moods. To think of a patient’s behaviour as normal in light of what they had experienced made the wards more agreeable to staff and patients. Feeling safe and creating moments of gratitude, happiness and appreciation was considered a goal in itself:

Some of our patients may never be able to live fully independently in society. I [nurse] believe that being accepted and valued is a feeling that can be transferred and internalized to replace some of the body-based survival strategies that come into play in stressful moments. We try to replace the overwhelming vulnerability with strategies to increase the patient’s self-respect and self-confidence, and even if we only partially succeed in helping all the patients become independent and live for themselves, it is my opinion that the ability to show care for others is an extremely important resource in society as a whole. Nurse 9, ward 2.

In the nurses’ opinion, humility towards what one cannot understand, comprehend and change without involving the patient is essential in establishing a trustworthy and secure relationship with a person who has experienced serious and inhumane betrayal and that mutual respect is not possible without humanizing care and lowering the attention to losses and diagnoses.

## Discussion and comprehensive understanding

This study provides insight into nurses’ experiences related to the humanization of care in patients’ everyday lives in high-security forensic wards. The nurses included in this study highlighted the value of openness and responsiveness to patients’ lived experiences and individual patients’ trauma-related stressors. Attention to patients’ individual needs is essential for understanding their reactions and helping them avoid stress and confrontation. In addition, in strict environments, the nurses highlighted the need to spend time with patients, showing that they deserve to be respected and cared for like human beings.

The nurses in our study highlighted the importance of having a caring presence and attitude towards patients in everyday life activities in the ward. The purpose of the humanization of care is to see and listen to patients’ life stories at the human level and find ways to invite patients into human fellowships. This is in line with the results of Sanz-Osorio et al. (2023), who reported that humanization can be translated into steps towards equality and respect for patients who are more prone to aggressive and pathological behaviour. Additionally, Hörberg (2018) reported that a caring relationship is a prerequisite for understanding and meeting patients in forensic psychiatric wards as human beings and ensuring human fellowship.

Fosse et al. (2021) highlighted the importance of understanding how a person who experiences neglect and abuse in childhood may develop sensitivity and the need to be in control and become skilled in observing others. In our study, the nurses focused on having a respectful attitude and honest and open-minded interest towards patients to gain insight into individual patients’ reflections on their situations and lived experiences. The nurses also focused on how patients present their intentions in complex situations that might involve danger. This was a long-term but very important process with respect to both the nurses and patients sharing the same world and creating an honest relationship. Kirkengen (2017) explained that persons who have experienced being let down and not being listened to or included develop an awareness and sensitivity in reading other people’s intentions, and it takes time for them to trust others.

In our study, the nurses highlighted situated supervision to help patients gain an understanding of their own actions and feelings and to make them receptive to supervision and therapy. Fosse et al. (2021) explain that supervision requires an openness to and cocreated individualized treatment and caring programmes where patients are involved in decision—making concerning their situations. This is in line with the study by Sanz-Osorio et al. (2023), who reported that supervision must be performed coherently and in ways that give patients the needed experiences of shared reality. In addition, being guided by professionals who allow patients to be involved in their own process gives patients a feeling of being considered fellow human beings. The nurses in our study stated that involving patients in decision-making regarding their own situations gives patients a feeling of being seen, respected and listened to. Furthermore, the experience of feeling equal might help patients find new and constructive ways of relating to other people.

The nurses highlighted the importance of being present and interested in individual patients’ situations and needs. Other recent studies have reported that knowing about and being involved in patients’ lives requires that nurses feel safe and protected within a healthcare professional team (Norcross & Lambert, 2018; Oben, 2020; Sollied et al., 2023). Caring for patients within a strict security-regulated atmosphere may cause ethical and personal stress for the nurses involved, and this responsibility requires openness and close collaboration within professional healthcare teams to strengthen nurses’ beliefs in the quality of their own patient involvement (Söderberg et al., 2022; Sollied et al., 2023). Sollied et al. (2023) reported that collaboration and support from a professional healthcare team may increase nurses’ professional competence in seeing a human being beyond a violent appearance and diagnostic criteria.

The nurses in our study focused on the patients’ needs and recovery, where the nurses allowed the patients to investigate new ways of thinking and, at the same time, allowed the patients to guide the nurses in understanding the patients’ lives from their points of view. Oben (2020) stated that it is important to remind nurses that being chosen as a person in whom a patient can find rest and trust in is a gift. The nurses in our study highlighted that reflection with other team members with respect to their own appearance was essential for remaining professional and for trusting in their own interpretations and judgement of what is the best for patients.

Appreciation of the patients’ own qualities and a genuine interest in who the patients are as people and what their world looked like was highly valued. Focusing on the qualities of an intertwined and shared vulnerability in nurse—patient encounters may provide valuable insight regarding understanding each other’s strengths and shortcomings as fellow human beings, which may enhance patients’ recovery processes and self-understanding. Likewise, when nurses feel free to be authentic in caring and doing good, both patients and nurses can experience a caring and humanized practice (Hammarström et al., 2022; Sanz-Osorio et al., 2023; Sollied et al., 2023).

## Limitations

A limitation of the study is that data from 2013 were used. To ensure that the data are still relevant, four of the nurses who continue to work in the wards were consulted to ensure that the security routines and procedures have not undergone significant changes. The discussion and comprehensive understanding section presents our results with respect to those of recent relevant studies. In addition, we argue that qualitative research findings are positioned in a cultural context, and thus, the validity of older qualitative data must depend less on time itself and more on whether the cultural context and structure within forensic care persist or if there have been social changes.

Our findings are related to how nurses experience the essential meaning of providing humanistic care within restrictive environments in forensic care units. Forensic psychiatry is governed and connected to how law and psychiatry are founded in national frameworks that differ across countries, which may influence how nurses practice patient care.

## Conclusion

This study provides insight into nurses’ experiences related to the humanization of care in high-security forensic wards. Nurses’ presence and caring attitudes in everyday activities in forensic psychiatric care wards provide insights into individual patients’ lived experiences, vulnerability, fragility and tolerance limits. The ability to see and listen to patients at the human level and meet and respect patients in human fellowships is essential for the humanization of care. In addition, the nurses in our study highlighted the need for nurses to feel safe and closely collaborate within professional healthcare teams.

## Clinical implications and suggestions for future research

We believe that the insights and knowledge gained from this study may be beneficial for healthcare practice, education, and future research.

We suggest that healthcare professionals can influence patients through interactions in daily life activities and should be valued as important support for patient recovery because of their familiarity with individual patients’ tolerance limits and lived experiences. This must be given attention, as clinical as well as psychological and social knowledge can be used to promote a humanizing atmosphere that lowers the risk of violence and strengthens the recovery process by empowering forensic patients.

Increased emphasis needs to be placed on the humanization of care in high-security forensic wards and improving nurses’ presence and caring attitudes in the education system.

## Data Availability

Due to the sensitive nature of the questions asked in this study, participants were assured that their raw data would remain confidential and would not be shared.
